# Case of Mental Excitement Allayed by Music

**Published:** 1846-08

**Authors:** 


### ARTICLE IV.

### *Case of Mental Excitement allayed by Music.*

Mr. S——, a young man 17 years of age, of a strongly marked nervous temperament, and rather delicate constitution, had a severe attack of remittent fever attended with cerebral excitement, and followed by nervousness and general debility.

During convalescence, being fond of books, he commenced reading some poetical work, with which he became so much interested, as to continue its perusal six or eight hours, with little or no intermission. Nervous irritability and general febrile excitement, were, as might have been expected, almost the immediate consequences of this imprudent mental effort, and in a few hours after, a state of delirium, with symptoms very similar in every respect to mania à potu, rendered the case truly alarming.

The symptoms indicated, as it seemed, the prompt use of narcotics. Morphine was therefore given in doses gradually increased till at the end of 48 hours, 3 gr. at a time, with strong laudanum injections had been administered. This treatment seeming to have little or no effect, was abandoned, and other means, such as baths, counter irritants, stimulants, &c. &c., resorted to, with but slight amelioration of the alarming symptoms.

The patient had now continued in this state three days and nights, without sleep, and with little or no food. Pulse much of the time 120. Countenance anxious and sunken, presenting every appearance in fact, of approaching final prostration.

Of the means above mentioned, the administration of brandy, in often repeated and large doses, seemed to act most favorably and effectually. Under its use the pulse came down to about 100. The patient also became more quiet, and manifested a slight disposition to sleep.

At this time, it was suggested by the father, that his son had always manifested a remarkable fondness for music, and that when a child, sleep had often been produced by it.

A violin player was accordingly sent for, and the effect of his art tested upon the patient, with the most remarkable and immediate favorable effects. The nervous excitement began to abate at the sound of the fiddle, and in a very short time, the patient was in a sound sleep, from which he awoke in an hour or two much refreshed and nearly rational.

By continuing the brandy, and when nervous excitement began to manifest itself, an occasional quietus from the fiddle, this singular state of mental excitement was, in a few days, entirely and permanently subdued. W. B. H.

